# Antioxidant Ability and Mechanism of Rhizoma *Atractylodes macrocephala*

**DOI:** 10.3390/molecules171113457

**Published:** 2012-11-13

**Authors:** Xican Li, Jian Lin, Weijuan Han, Wenqiong Mai, Li Wang, Qiang Li, Miaofang Lin, Mingsong Bai, Lishan Zhang, Dongfeng Chen

**Affiliations:** 1 School of Chinese Herbal Medicine, Guangzhou University of Chinese Medicine, Guangzhou 510006, China; 2 School of Basic Medicine, Guangzhou University of Chinese Medicine, Guangzhou 510006, China

**Keywords:** Rhizoma *Atractylodes macrocephala*, baizhu, antioxidant activity, metal chelating, radical-scavening, phenolic acid

## Abstract

Rhizoma *Atractylodes macrocephala* (AM) has been used in Traditional Chinese Medicine (TCM) for about 2,000 years. In the study, we firstly determined the antioxidant levels of five AM extracts by •OH-scavenging, •O_2_^−^-scavenging, Fe^2+^-chelating, Cu^2+^-chelating, DPPH·-scavenging, and ABTS^+^·-scavenging assays. After measurement of the chemical contents in five AM extracts, we quantitatively analyzed the correlations between antioxidant levels and chemical contents. It was observed that total phenolics and total flavonoids had significant positive correlations with antioxidant levels (R = 0.685 and 0.479, respectively). In contrast, total sugars and total saponins presented lower correlations with antioxidant levels (R = −0.272 and 0.244, respectively). It means that antioxidant activity of AM should be attributed to total phenolics (including phenolic acids and flavonoids), and not total sugars and total saponins. Further analysis indicated that phenolic acids exhibited higher R values with radical-scavenging assays (R = 0.32–1.00), while flavonoids showed higher R values with metal-chelating assays (R= 0.86 and 0.90). In conclusion, AM exerts its antioxidant effect through metal-chelating, and radical-scavenging which is via donating hydrogen atom and donating electron. Its metal-chelating may result from flavonoids, while its radical-scavenging can be attributed to phenolic acids, especially caffeic acid, ferulic acid, and protocatechuic acid.

## 1. Introduction

It is well known that excessive ROS (reactive oxygen species) can oxidatively damage cellular structures, lead to deleterious biological consequences, including mutation [1], cell death [2], carcinogenesis [2], and aging [2]. Over the past decades, an intensive search for potential therapeutic agents for oxidative damage has been carried out in medicinal plants [3], especially Chinese medicinal herbals.

As a typical Chinese herbal medicine for replenishing *qi*, Rhizoma *Atractylodes macrocephala* (AM, baizhu in Chinese) has been used in traditional Chinese medicine (TCM) for about 2,000 years [4,5]. Modern medicine has indicated that it possesses various pharmacological effects involving antioxidant ability, such as anti-mutation [6], anti-tumor [7], anti-aging [8], promotion of cellular growth and differentiation [9], upregulation of the expression of SOD (superxide dismutase) & GSH-PX (glutathione peroxidase) of mouse [10,11], and so on, but no study of the antioxidant properties of AM has been reported so far. Therefore, the aim of this study was to systematically investigate the antioxidant ability of AM, then further discuss the antioxidant mechanism.

## 2. Results and Discussion

As an important form of ROS, hydroxyl radical (•OH), is generated in the human body via the Fenton reaction (Equation 1):

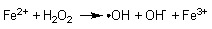
(1)


Since •OH radicals are extremele reactive, they can easily damage proteins, lipids, sugars and DNA. For example, •OH attacks deoxyribose, which is the backbone of DNA, to generate malondialdehyde (MDA) (Equation 2). MDA then combines 2-thiobarbituric acid (TBA) to yield TBARS (thiobarbituric acid reactive substances) which presents a maximum absorbance at 530 nm (Equation 3) [12].

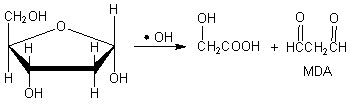
(2)

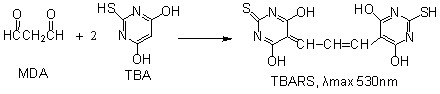
(3)


**Figure 1:**
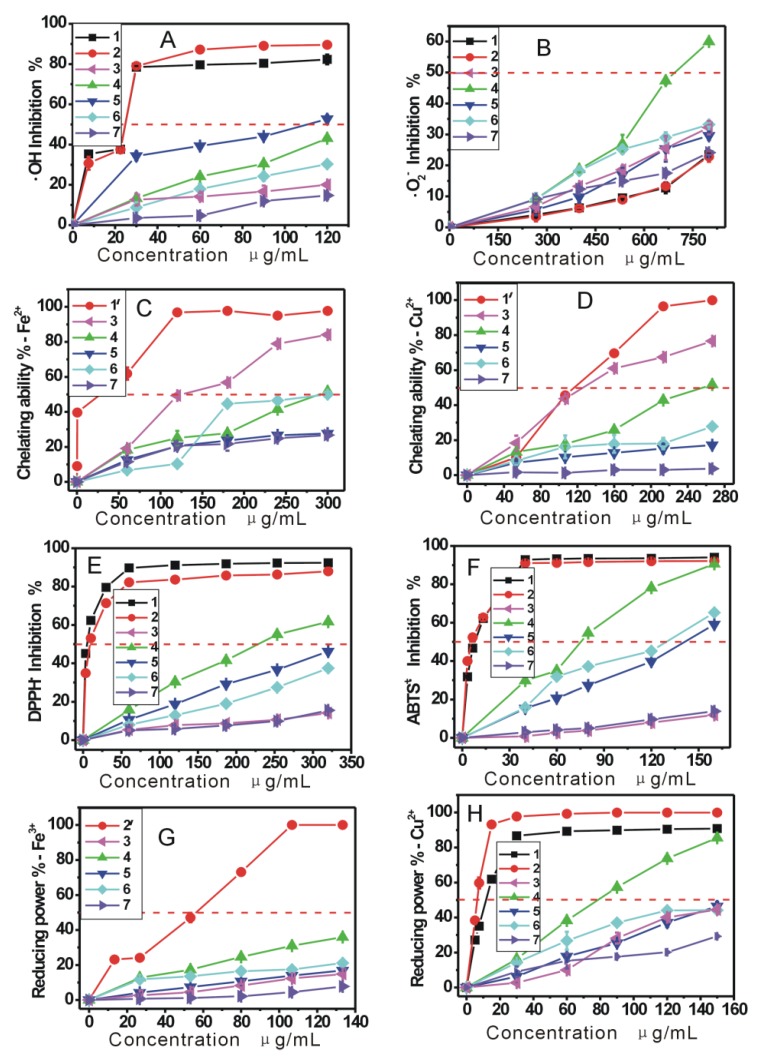
The dose response curves of five extracts in the antioxidant assays: (**A**) •OH scavenging; (**B**) •O_2_^−^ scavenging; (**C**) Fe^2+^-chelating; (**D**) Cu^2+^-chelating; (**E**) DPPH• scavenging; (**F**) ABTS^+^• scavenging; (**G**) Fe^3+^-reducing; (**H**) Cu^2+^-reducing. **1**—Trolox; **1'**—Sodium citrate; **2**—BHA (butylated hydroxyanisole); **2'**—GSH (glutathione); **3**—PEAM (petroleum ether extract of Rhizoma *Atractylodes macrocephala*); **4**—EAAM (ethyl acetate extract of Rhizoma *Atractylodes macrocephala*); **5**—AEAM (absolute ethanol extract of Rhizoma *Atractylodes macrocephala*); **6**—95EAM (95% ethanol extract of Rhizoma *Atractylodes macrocephala*); **7**—WAM (water extract of Rhizoma *Atractylodes macrocephala*).

Hence, the value of A_532nm_ can reflect the amount of •OH radicals. If an antioxidant sample is added, A_532nm_ value will decrease, suggesting that some •OH radicals are scavenged by the antioxidant. This is the principle of deoxyribose degradation assay used in the study. Using this deoxyribose degradation assay, we measured the •OH radical-scavenging ability of AM. As shown in Figure 1A five AM extracts scavenged •OH radicals in a dose-dependent manner.

Besides •OH, superoxide anion (•O_2_^−^) is also regarded as an important form of ROS in living cells. Although •O_2_^−^ is much weaker than •OH, however, it is able to directly attack DNA and lipids [13], or transform into •OH via the Haber-Weiss reaction (Equation 4), to damage biomolecules [14]:

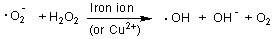
(4)


In the study, the •O_2_^−^ -scavenging ability of AM was determined at physiological pH 7.4 [15]. Figure 1B shows that the •O_2_^−^-scavenging percentages of the five AM extracts increased in a concentration-dependent manner.

As indicated in Equations 1 and 4, transition metals (especially Fe and Cu) can catalyze the generation of •O_2_^−^ and •OH radicals. The metal-chelating ability of AM was therefore explored in the study. The dose-response curves (Figure 1C,D) confirmed that the five AM extracts presented effective metal chelating abilities. The fact that AM can bind the Fe^2+^ and Cu^2+^, suggests that metal-chelating may be one of mechanisms used to scavenge •OH or •O_2_^−^.

In order to confirm whether AM can directly scavenge radicals, the five AM extracts were further determined using the DPPH and ABTS assays. As seen in Figure 1E,F, the five extracts can effectively eliminate DPPH• and ABTS•^+^ radicals. Since the generation of both these radicals don’t involve the transition metal catalysis, direct radical-scavenging can be considered as another mechanism for AM to scavenge •OH or •O_2_^−^.

The previous studies have demonstrated that DPPH· may be scavenged by an antioxidant through donation of a hydrogen atom (H•) to form a stable DPPH-H molecule [16,17,18]. For example, the mechanism of DPPH• scavenging by ferulic acid can be proposed to occur as shown in Equation 5:

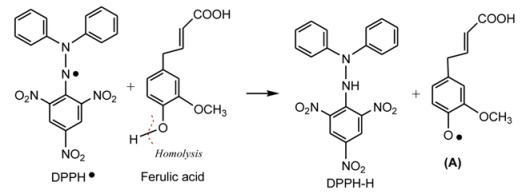
(5)


Unlike DPPH• scavenging, ABTS•^+^ scavenging is considered an electron (e) transfer reaction [19]. For example, the proposed reaction of ferulic acid and ABTS·^+^ can be briefly illustrated by Equation 6:

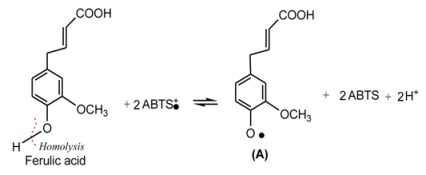
(6)


In a word, the five AM extracts can effectively scavenge DPPH• and ABTS^+^• radicals, suggesting that AM exerts radical-scavenging action by donating hydrogen atoms (H•) and electrons (e).

Phytochemical studies indicated that AM mainly contained volatile oil, polysaccharides, flavonoids, saponins, and total phenolics [20,21]. From its volatile oil, atractylenolide I, II, and III could be obtained by eluting with petroleum ether-diethyl ether [22,23]. From the hot aqueous or alcoholic extracts of AM, various sugars and glycosides were isolated, such as saponins, mannose, fructose, and inulin [20,24]. Flavonoids, however, could be extracted from AM by ethanol [25]. Taken together, the volatile oil and atractylenolides mainly exist in petroleum ether (non or lower polar solvent) extracts, while sugars and glycosides generally occur in water or hot alcohol extracts, and flavonoids could be enriched by ethanol extraction.

As shown in Figure 1, the results of •O_2_^−^ assay, Fe^2+^-chelating, Cu^2+^-chelating, DPPH• assay, ABTS^+^• assay, Fe^3+^-reducing, and Cu^2+^-reducing assay were generally similar. According to the IC_50_ values (Table 1), the order of antioxidant levels of the five AM extracts was EAAM > AEAM ≈ 95EAM > PEAM > WAM in the five assays. This is because the bioactive components relevant to antioxidant possess mild polarity, so they can be easily extract by mild polarity ethyl acetate. These bioactive components mainly included phenolic acids and flavonoids. In the water extract, the enriched components were saccharides and saponins which possess weak antioxidant properties. Therefore, EAAM exhibited the strongest abilities and WAM presented the weakest abilities in the five antioxidant assays. 

Unlike the five antioxidant assays above, the •OH assay did not present so significant differences between the antioxidant levels of the AM extracts (Figure 1A). In fact, •OH has extremely high reactivity and can react with any substance. Hence, the hydroxyl-scavenging abilities cannot be greatly affected by the types or contents of components, and the difference between the antioxidant levels of the AM extracts was the smallest.

In order to further confirm which chemical component could be responsible for the antioxidant of AM, we quantitatively analyzed the correlations (R values) between antioxidant levels and chemical contents in AM extracts. In the study, a spectrophotometric method was used to measure the relevant chemical contents in the five AM extracts, including total phenolics, total saponins and total sugars (Table 2 & Supplementary Information Part 2). On the other hand, since the 1/IC_50_ value (not IC_50_ value) showed a parallelism with antioxidant activity, 1/IC_50_ values were then used for evaluating antioxidant levels in the study (Supplementary Information Part 3). On this basis, the correlation graphs were plotted (Supplementary Information Part 6) and the R values were calculated (Table 3). The results suggested that total phenolics had significant positive correlations between antioxidant levels (R = 0.45~0.90, the average R value was 0.685), while total sugars and total saponins possessed lower or negative correlations (the average values of R were −0.272 and 0.244, respectively). This implies that the antioxidant activity of AM should be attributed to the presence of total phenolics, not of total sugars and total saponins.

Earlier studies have shown that AM polysaccharides also exhibited antioxidant ability [26,27]. However, the IC_50_ values of polysaccharides in DPPH assay (>8 mg/mL) [27] far exceeded those of total phenolics (239.42–1444.26 μg/mL, Table 1), suggesting the antioxidant activity of polysaccharides was actually much weaker than total phenolics. It can be inferred that polysaccharides don’t effectively exert the antioxidant action when they are used along with phenolics in AM or AM extracts.

**Table 1:** The IC_50_ values of five extracts from Rhizoma *Atractylodes macrocephala* (µg/mL).

	PEAM	EAAM	AEAM	95EAM	WAM	Positive controls	
						Trolox	BHA
•OH scavenging	419.62 ± 9.48 ^g^	140.39 ± 1.26 ^d^	104.97 ± 2.64^c^	193.70 ± 4.71 ^e^	357.52 ± 4.31 ^f^	36.67 ± 0.67 ^b^	27.95 ± 0.39 ^a^
•O_2_^−^ scavenging	1227.26 ± 41.27 ^b^	699.29 ± 6.30 ^a^	1115.68 ± 170.20 ^b^	1079.10 ± 26.79 ^b^	2191.70 ± 58.08 ^c^	2118.20 ± 30.01 ^c^	2178.82 ± 18.02 ^c^
Fe^2+^-chelating	142.70 ± 1.92 ^b^	303.70 ± 4.86 ^c^	543.86 ± 17.94 ^d^	305.87 ± 1.33 ^c^	697.69 ± 36.96 ^e^	26.32 ± 2.72 ^*,a^	ND
Cu^2+^-chelating	134.91 ± 3.92 ^b^	255.94 ± 1.04 ^c^	1025.38 ± 75.44 ^e^	507.61 ± 1.98 ^d^	7047.13 ± 895.31 ^f^	116.77 ± 1.58 ^*,a^	ND
DPPH• scavenging	1444.26 ± 68.47 ^g^	239.42 ± 0.64 ^c^	347.72 ± 0.60 ^d^	446.84 ± 11.42 ^e^	1077.70 ± 19.35 ^f^	4.10 ± 0.21 ^a^	11.98 ± 0.49 ^b^
ABTS^+^• scavenging	534.68 ± 11.30 ^f^	78.25 ± 0.30 ^c^	140.00 ± 0.16 ^e^	122.34 ± 0.83 ^d^	471.41 ± 36.91 ^f^	8.84 ± 0.14 ^b^	6.99 ± 0.05 ^a^
Fe^3+^-reducing	442.25 ± 10.15 ^c^	197.41 ± 1.53 ^b^	411.19 ± 9.11 ^c^	420.12 ± 2.63 ^c^	667.04 ± 20.58 ^d^	51.84 ± 0.99 ^**,a^	ND
Fe^3+^-reducing	137.59 ± 2.18 ^d^	83.24 ± 0.59 ^c^	160.86 ± 8.42 ^e^	133.75 ± 0.28 ^d^	277.42 ± 19.97 ^f^	12.44 ± 0.17 ^b^	6.54 ± 0.25 ^a^

IC_50_ value is defined as the concentration of 50% effect percentage and expressed as Mean ± SD (n = 3). Means values with different superscripts in the same row are significantly different (*p* < 0.05), while with same superscripts are not signifiacntly different (*p* < 0.05). ^*^ The positive control is sodium citrate. ^**^ The positive control is GSH (glutathione). BHA, butylated hydroxyanisole. PEAM, petroleum ether extract of Rhizoma *Atractylodes macrocephala*. EAAM, ethyl acetate extract of Rhizoma *Atractylodes macrocephala*. AEAM, absolute ethanol extract of Rhizoma *Atractylodes macrocephala*. 95EAM, 95% ethanol extract of Rhizoma *Atractylodes macrocephala*. WAM, water extract of Rhizoma *Atractylodes macrocephala*. ND, cannot be detected.

**Table 2:** The chemical contents of five extracts from Rhizoma *Atractylodes macrocephala*.

	PEAM	EAAM	AEAM	95EAM	WAM
Total phenolics (mg Pyr./g)	15.32 ± 0.00 ^d^	21.03 ± 0.052 ^e^	13.61 ± 0.052 ^c^	10.01 ± 0.026 ^b^	2.43 ± 0.045 ^a^
Total flavonoids (mg rutin/g)	3.36 ± 0.0064 ^d^	2.88 ± 0.011 ^c^	0.50 ± 0.00 ^b^	2.09 ± 0.00 ^c^	0.026 ± 0.064 ^a^
Total sugar (mg glucose/g)	1.10 ± 0.016 ^a^	1.54 ± 0.0090 ^a^	4.97 ± 0.00 ^c^	4.99 ± 0.016 ^c^	3.56 ± 0.0066 ^b^
Total saponin (mg Ole./g)	14.98 ± 0.021 ^d^	6.08 ± 0.11 ^c^	5.08 ± 0.20 ^c^	2.48 ± 0.19 ^b^	0.026 ± 0.064 ^a^
Caffeic acid (peak area)	5530.33 ± 684.22 ^b^	15863.98 ± 1254.02 ^d^	6466.64 ± 655.75 ^c^	5990.33 ± 94.99 ^b^	4333.34 ± 194.72 ^a^
Ferulic acid (peak area)	7768.53 ± 584.85 ^b^	22358.69 ± 2001.20 ^d^	8878.73 ± 784.7 ^c^	7968.53 ± 324.90 ^b^	5013.33 ± 424.87 ^a^
Protocatechuic aicd (peak area)	0.00 ^a^	463523 ± 44277.3 ^c^	0.00 ^a^	51570.5 ± 714.89 ^b^	0.00 ^a^

Each value is expressed as Mean ± SD (n = 3). Means values with different superscripts in the same row are significantly different (*p <* 0.05), while with same superscripts are not signifiacntly different (*p <* 0.05). PEAM, petroleum ether extract of Rhizoma *Atractylodes macrocephala*. EAAM, ethyl acetate extract of Rhizoma *Atractylodes macrocephala*. AEAM, absolute ethanol extract of Rhizoma *Atractylodes macrocephala*. 95EAM, 95% ethanol extract of Rhizoma *Atractylodes macrocephala*. WAM, water extract of Rhizoma *Atractylodes macrocephala*. Ole., oleanolic. Pyr., pryrogallol.

It was reported that total phenolics in AM generally include flavonoids [28] and phenolic acids [29]. Therefore, we estimated the total flavonoids of the five AM extracts. Similarly, the correlation graphs (Supplementary Information Part 6) between total flavonoids and 1/IC_50_ values were drawn to calculate the R values. The average R value (0.479) in Table 3 revealed that flavonoids might also be the relevant antioxidant components of AM. However, the individual R values varied with the antioxidant assays. In general, the R values in radical-scavenging assays (R = −0.21–0.74) were much lower than those in the chelating assays (R = 0.86 and 0.90). This means that flavonoids may be responsible for the chelating abilities of AM. Despite the fact individual flavonoids have not been isolated from AM so far, however, we believe that these flavonoids may contain *ortho*-dihydroxyl groups in their molecules, because chelating ability in antioxidant molecules usually results from the presence of such *ortho*-dihydroxyls [30].

**Table 3:** The R (correlation coefficient) values between chemical contents and antioxidant levels.

	Total phenolics	Total flavonoids	Total sugars	Total saponins	Caffeic acid	Ferulic acid	PCA
•OH assay	0.45	−0.21	0.43	−0.21	0.44	0.45	0.32
•O_2_^−^ assay	0.90	0.60	−0.31	0.22	0.92	0.93	0.87
Fe^2+^-chelating	0.46	0.86	−0.68	0.92	0.00092	0.040	−0.023
Cu^2+^-chelating	0.62	0.90	−0.81	0.94	0.22	0.26	0.18
DPPH· assay	0.66	0.14	0.066	−0.20	0.85	0.85	0.80
ABTS^+^• assay	0.65	0.26	0.055	−0.21	0.85	0.85	0.82
Fe^3+^-reducing	0.84	0.54	−0.45	0.16	0.99	1.00	0.97
Cu^2+^-reducing	0.90	0.74	−0.48	0.33	0.92	0.93	0.88
**Average**	**0.685**	**0.479**	**−0.272**	**0.244**	**0.649**	**0.664**	**0.602**

PCA, protocatechuic acid.

As for another type of total phenolics, phenolic acidd were also analyzed in the study. The HPLC analysis identified three phenolic acids in AM extracts, including caffeic acid, ferulic acid and protocatechuic acid. Further analysis indicated that three phenolic acids were of higher average R values (0.649, 0.664, and 0.602, respectively) with antioxidant levels, so these three phenolic acids can also be regarded as relevant antioxidant components of AM. However, contrary to the flavonoids, phenolic acids exhibited higher R values with radical-scavenging assays (R = 0.32–1.00), but lower R values with chelating assays (R = −0.023–0.26) (Table 3). This means that three phenolic acids may act as radical-scavengers and not metal-chelators in AM. 

## 3. Experimental

### 3.1. Plant Material

Rhizoma *Atractylodes macrocephala* was purchased from Yanghe Pharmacy of Guangzhou University of Chinese Medicine (Guangzhou, China), and authenticated by Professor Shuhui Tan. A voucher specimen was deposited in our laboratory.

### 3.2. Chemicals

Trolox (±-6-hydroxyl-2,5,7,8-tetramethlyhromane-2-carboxylic acid), ferrozin [3-(2-pyridyl)-5,6-bis(4-phenylsulfonic acid)-1,2,4-triazine], BHA (butylated hydroxyanisole), DPPH• (1,1-diphenyl-2-picrylhydrazyl radical), neocuproine (2,9-dimethyl-1,10-phenanthroline), pyrogallol, murexide (5,5′-nitrilodibarbituric acid monoammonium salt), and protocatechuic acid were purchased from Sigma Aldrich Trading Co. (Shanghai, China); ABTS diammonium salt [2,2′-azino-bis(3-ethyl-benzothiazoline-6-sulfonic acid diammonium salt)], D-2-deoxyribose, and GSH (glutathione) were obtained from Amresco Co. (Solon, OH, USA); Ferulic acid and caffeic acid were purchased from National Institute for the Control of Pharmaceutical and Biological Products (Beijing, China). Acetonitrile, methanol and water were of HPLC grade. All other chemicals used were analytical grade.

### 3.3. Preparation of Five AM Extracts

**Figure 2:**
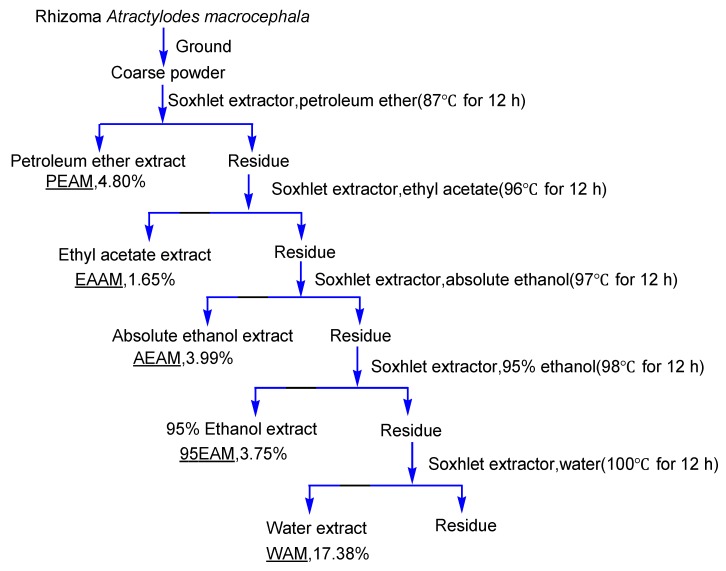
The preparation of five extracts from Rhizoma *Atractylodes macrocephala*.

The dried Rhizoma *Atractylodes macrocephala* was ground to a coarse powder then extracted in sequence with petroleum ether (60–90), ethyl acetate, ethanol, 95% ethanol and water by Soxhlet extractor for 12 h (Figure 2). Extracts were filtered using a Büchner funnel and Xinhua No. 1 filter paper. Each filtrate was concentrated to dryness under reduced pressure at 60 °C using a rotary evaporator.

### 3.4. Hydroxyl (•OH) Radical-Scavenging Assay

The scavenging activity on the hydroxyl radical was investigated by the deoxyribose method [30], with some modifications. Our preliminary experiments demonstrated that most organic solvents especially alcohol can promote the inhibition percentage value. Hence, the inhibition of the hydroxyl radical was evaluated as follows: all test samples were first dissolved in methanol at 4 mg/mL. An aliquot of sample solution (12–48 μL) was brought to a tube, then the sample solution in tube was evaporated to dryness (water bath, 60 °C). The sample residue was mixed with 400 μL phosphate buffer (0.2 mol/L, pH 7.4). Subsequently, 50 μL deoxyribose (50 mmol/L), 50 μL H_2_O_2_ (50 mmol/L), 50 μL FeCl_3_ (3.2 mmol/L) and 50 μL Na_2_EDTA (1 mmol/L) were added. The reaction was initiated by mixing 50 μL ascorbic acid (1.8 mmol/L) and the total volume of the reaction mixture was adjusted to 800 μL with buffer. After incubation at 50 °C for 20 min, the reaction was terminated by 250 μL trichloroacetic acid (10%, w/w).

The color was then developed by addition of 150 μL TBA (1g/20 mL, in 1.25% NaOH aqueous solution) and heated in an oven at 105 °C for 15 min. The mixture was cooled and absorbance was measured at 530 nm against the buffer (as blank). The inhibition percentage for ·OH is expressed as follows:

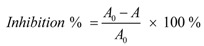

where, *A_0_* is the *A_532nm_* of mixture without sample, and *A* is the *A_532nm_* of the mixture with sample.

### 3.5. Superoxide Anion (•O_2_^−^) Radical-Scavenging Assay

Measurement of superoxide anion (•O_2_^−^) scavenging activity was based on our method [15]. Briefly, the sample was dissolved in ethanol at 8 mg/mL. The sample solution (*x* μL, where *x* = 0, 100, 150, 200, 250, and 300 μL) was mixed with 2920-*x* μL Tris-HCl buffer (0.05mol/L, pH 7.4) containing EDTA (1 mmol/L). When 80 μL pyrogallol (60 mmol/L in 1 mmol/L HCl) was added, the mixture was shaken at room temperature immediately. The absorbance at 325 nm of the mixture was measured (Unico 2100, Shanghai, China) against the Tris-HCl buffer as blank every 30 s for 5 min. The •O_2_^−^ scavenging ability was calculated as:

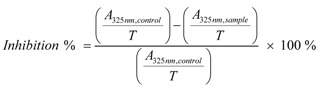

where, *ΔA_325nm, control_* is the increase in *A_325nm_* of the mixture without the sample and *ΔA_325nm, sample_* is that with the sample; *T* = 5 min. The experiment temperature was 37 °C.

### 3.6. Fe^2+^-Chelating Assay

The Fe^2+^ chelating activities of five AM extracts were estimated by the method as described by Li [31]. Briefly, *x* μL sample solutions (2 mg/mL, *x* = 0, 30, 60, 90, 120, and 150) were added to 100 μL FeCl_2_ aqueous solutions (250 μmol/L). The reaction was initiated by the addition of 150 μL ferrozine aqueous solutions (500 μmol/L) and the total volume of the reaction mixture was adjusted to 1,000 μL with methanol. Then, the mixture was shaken vigorously and stood at room temperature for 10 min. Absorbance of the solution was measured spectrophotometrically at 562 nm (Unico 2100). The percentage of chelating effect on Fe^2+^ was calculated by the following formula:

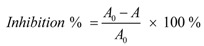

where *A_0_* is the absorbance without sample, and *A* is the absorbance with sample.

### 3.7. Cu^2+^-Chelating Assay

The Cu^2+^-chelating activities of the five AM extracts were measured by a complexometric method using murexide [31]. Briefly, murexide solution (1.2 mL, 0.25 mmol/L) and CuSO_4_ aqueous solution (60 μL, 20 mmol/L) were added to hexamine HCl buffer (pH 5.0, 30 mmol/L) containing 30 mmol/L KCl. After incubation for 1 min at room temperature, 40–200 μL sample solutions (2 mg/mL in methanol) were added. The final volume was adjusted to 1,500 μL with methanol. Then, the mixture was shaken vigorously and left at room temperature for 10 min. Absorbance of the solution was then measured spectrophotometrically at 485 nm and 520 nm (Unico 2100). The absorbance ratio (*A_485_/A_520_*) reflected the free Cu^2+^ content. Therefore, the percentage of cupric chelating effect was calculated by the following formula:

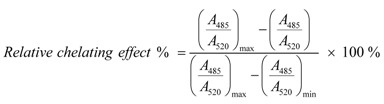

where 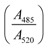 is the absorbance ratio of the sample, while 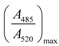 is the maximum absorbance ratio and 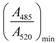 is the minimum absorbance ratio in the test.

### 3.8. DPPH• Radical-Scavenging Assay

DPPH• radical-scavenging activity was determined as described [32]. Briefly, 1 mL DPPH• ethanolic solutions (0.1 mmol/L) were mixed with 0.5 mL sample alcoholic solutions (0.18–0.96 mg/mL). The mixtures were kept at room temperature for 30 min, and then measured with a spectrophotometer (Unico 2100, Shanghai, China) at 519 nm. The DPPH• inhibition percentages were calculated:

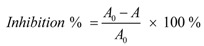

where *A* is the absorbance with samples, while *A_0_* is the absorbance without samples. Trolox and BHA were used as the positive controls.

### 3.9. ABTS^+^• Radical-Scavenging Assay

ABTS^+^• scavenging activity was measured as described [32] with some modifications. The ABTS^+^• was produced by mixing 0.2 mL ABTS diammonium salt aqueous solution (7.4 mmol/L) with 0.2 mL K_2_S_2_O_8_ aqueous solution (2.6 mmol/L). The mixture was kept in the dark at room temperature for 12 h to allow completion of ABTS^+^• generation. Before usage, it was diluted with 95% ethanol (about 1:50) so that its absorbance at 734 nm was 0.70 ± 0.02. Then, 1.2 mL diluted ABTS^+^• reagents were brought to 0.3 mL sample ethanolic solutions (0.2–0.8 mg/mL). After incubation for 6 min, the absorbance at 734 nm was read on a spectrophotometer (Unico 2100, Shanghai, China). The percentage inhibition was calculated as:

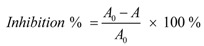

where *A_0_* is the absorbance of the mixture without sample, *A* is the absorbance of the mixture with sample (or positive control).

### 3.10. Fe^3+^-Reducing Power Assay

Ferric cyanide (Fe^3+^) reducing power was determined according to the method of Oyaizu [33] as described by Li *et al*. [32]. In brief, *x* μL sample methanolic solution (4 mg/mL, *x* = 0, 20, 40, 60, 80, and 100) was mixed with (500 − *x*) μL Na_2_HPO_4_/KH_2_PO_4_ buffer (0.2 mol/L, pH 6.6) and 250 μL K_3_Fe(CN)_6_ aqueous solution (1 g/100 mL). After the mixture was incubated at 50 °C for 20 min, it was added by 250 μL trichloroacetic acid (10 g/100 mL in distilled water) then centrifuged at 3500 rpm for 10 min. As soon as 400 μL supernatant was mixed with 400 μL FeCl_3_ (0.1 g/100 mL in distilled water), the timer was started. At 90 s, absorbance of the mixture was read at 700 nm (Unico 2100, Shanghai, China). Samples were analyzed in groups of three, and when the analysis of one group has finished, the next group of three samples were mixed with FeCl_3_ to avoid oxidization by air. The relative reducing ability of the sample was calculated by using the formula:

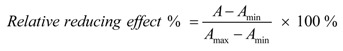



Here, *A*_*max*_ is the maximum absorbance and *A*_*min*_ is the minimum absorbance in the test. *A* is the absorbance of sample.

### 3.11. Cu^2+^-Reducing Power Assay

The cupric ions (Cu^2+^) reducing capacity was determined by the method [34], with minor modifications. Briefly, CuSO_4_ aqueous solution (125 μL, 0.01 mol/L), neocuproine ethanolic solution (125 μL, 7.5 mmol/L) and (750 − *x*) μL CH_3_COONH_4_ buffer solution (0.1 mol/L, pH 7.5) were brought to test tubes with different volumes of samples (1 mg/mL, *x* = 30–150 μL). Then, the total volume was adjusted to 1,000 μL with the buffer and mixed vigorously. Absorbance against a buffer blank was measured at 450 nm after 30 min (Unico 2100). The relative reducing power of the sample as compared with the maximum absorbance, was calculated by the formula:

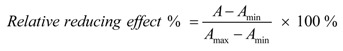

where, *A_max_* is the maximum absorbance at 450 nm and *A_min_* is the minimum absorbance in the test. *A* is the absorbance of sample.

### 3.12. Determination of Total Phenolics

The total phenolics contents of the five AM extracts were determined using a modified Folin-Ciocalteu colorimetric method [35]. Briefly, methanolic sample solution (0.5 mL, 1 mg/mL) was added to Folin-Ciocalteu reagent (0.5 mL, 0.25 mol/L). The mixture was left standing for 3 min, followed by the addition of Na_2_CO_3_ aqueous solution (1.0 mL, 15%, w/w). After incubation at ambient temperature for 30 min, the mixture was centrifuged at 3,500 rpm for 3 min. The supernatant was measured using a spectrophotometer (Unico 2100) at 760 nm. The standard curve was prepared using different concentrations of pyrogallol and the results were expressed as pyrogallol equivalents in milligrams per gram extract.

### 3.13. Determination of Total Flavonoids

Total flavonoids contents of the five AM extracts were measured using the NaNO_2_-Al(NO_3_)_3_ method with minor modifications [35]. In brief, methanolic sample solution (1 mL, 10 mg/mL) was mixed with NaNO_2_ aqueous solution (0.15 mL, 5%, w/w). The mixture stood at ambient temperature for 6 min, followed by the addition of Al(NO_3_)_3_ aqueous solution (0.15 mL, 10%, w/w). After incubation at ambient temperature for 6 min, NaOH aqueous solution (2 mL, 4%, w/w) was added to the mixture whicj was then adjusted to 5 mL with distilled water. The absorbance was read at 508 nm on a spectrophotometer (Unico 2100). The standard curve was prepared using different concentrations of quercetin and the results were also expressed as quercetin in milligrams per gram extract.

### 3.14. Determination of Total Sugars

The total sugars content was evaluated according to the phenol-sulfuric acid method [36]. An aliquot of sample solution (1 mL, 1 mg/mL) was placed in a test tube, and the volume was adjusted to 2 mL with distilled water. Then an aliquot of 5% phenol solution (1 mL) and concentrated sulfuric acid (5 mL) were added. After incubation for 20 min at room temperature, the reaction mixture was measured using a spectrophotometer (Unico 2100) at 490 nm. The standard curve was prepared using different concentrations of glucose and the results were expressed as glucose in milligrams per gram extract.

### 3.15. Determination of Total Saponins

The total saponins content was measured according to the method described in [36]. Methanolic sample solution (0.2 mL, 1 mg/mL) was taken in a test tube. After the methanol solvent was removed at 80 °C, an aliquot of vanillin-acetic acid solution (0.1 mL, 0.5%) and perchloric acid (0.4 mL) were added. The reaction mixture was incubated at 70 °C for 15 min, then cooled and diluted with acetic acid (1.25 mL). After 10 min, the absorbance of the diluted solution was measured at 540 nm (Unico 2100) against a blank control, which contained all reagents except for sample. Quantification was based on the standard curve for oleanolic acid (11–69 μg/mL). The results were expressed in milligrams of oleanolic acid equivalents per gram of extract.

### 3.16. HPLC Analysis

HPLC analysis was performed on a Syltech P510 system (Los Angeles, CA, USA), equipped with a Diamonsil C_18_ (250 mm × 4.6 mm, 5 μm) column (Dikma Co., Beijing, China). All samples were dissolved in methanol at 4 mg/mL and filtered using 0.45 μm filter.

**Figure 3:**
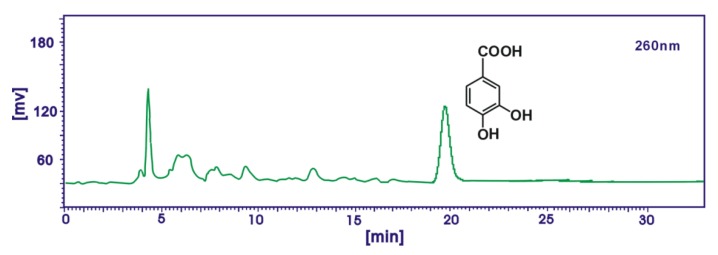
A typical HPLC profile of protocatechuic acid in the ethyl acetate extract of Rhizoma *Atractylodes macrocephala*. Syltech P510 system (Los Angeles, CA, USA), Dikma Diamonsil C_18_ (250 mm × 4.6 mm, 5 μm) column, 1.0 mL/min flow rate, methanol/acetonitrile/5% acetate acid (12:3:10), 10 μL injection, 60 nm wavelength.

**Figure 4:**
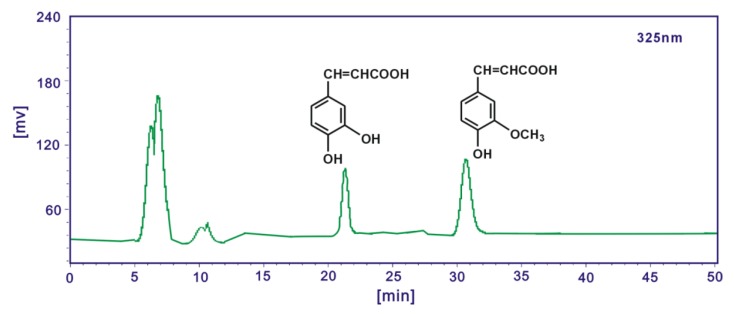
A typical HPLC profile of caffeic acid and ferulic acid in ethyl acetate extract of Rhizoma *Atractylodes macrocephala*. Syltech P510 system (Los Angeles, CA, USA), Dikma Diamonsil C_18_ (250 mm × 4.6 mm, 5 μm) column, methanol/acetonitrile/5% acetate acid (24:6:70), 0.5 mL/min flow rate, 10 μL injection, 325 nm wavelength.

In the analysis for caffeic acid and ferulic acid, the mobile phase was methanol/acetonitrile/5% acetate acid (24:6:70) and the flow rate was 0.5 mL/min, injection volume was 10 μL, detection wavelength was 325 nm. In the analysis for protocatechuic acid, the mobile phase was methanol/acetonitrile/5% acetate acid (12:3:10) and the flow rate was 1.0 mL/min, injection volume was 10 μL, detection wavelength was 260 nm. Caffeic acid, ferulic acid and protocatechuic acid in AM extracts were identified by the retention times and the peak areas were used to characterize the relative contents in the study (Figure 3 and Figure 4).

### 3.17. Statistical Analysis

Data are given as the mean ± SD of three measurements. The IC_50_ values were calculated by linear regression analysis. All linear regression in this paper was analyzed by Origin 6.0 professional software. Significant differences were performed using the T-test (*p <* 0.05). The analysis was performed using SPSS software (v.12, SPSS, Chicago, IL, USA).

## 4. Conclusions

Rhizoma *Atractylodes macrocephala* has effective antioxidant ability which may be responsible for its various pharmacological effects. It exerts its antioxidant effect through metal-chelation and radical-scavenging actions which occur via donation of hydrogen atoms (H•) and electrons (e). Its metal-chelation may result from its flavonoids, while its radical-scavenging is attributed to the presence of phenolic acids, especially caffeic acid, ferulic acid, and protocatechuic acid.

## Supplementary Materials

Supplementary materials can be accessed at: http://www.mdpi.com/1420-3049/17/11/13457/s1.
