# Supplementary material for: Antioxidant Ability and Mechanism of Rhizoma Atractylodes macrocephala
Source: Molecules. 2012 Nov 13;17(11):13457–72. doi: 10.3390/molecules171113457 (PMC6268131; doi:10.3390/molecules171113457)

Support Information

Part 1. Photos.

Figure S1-1. The plants of Rhizoma *Atractylodes macrocephala*.

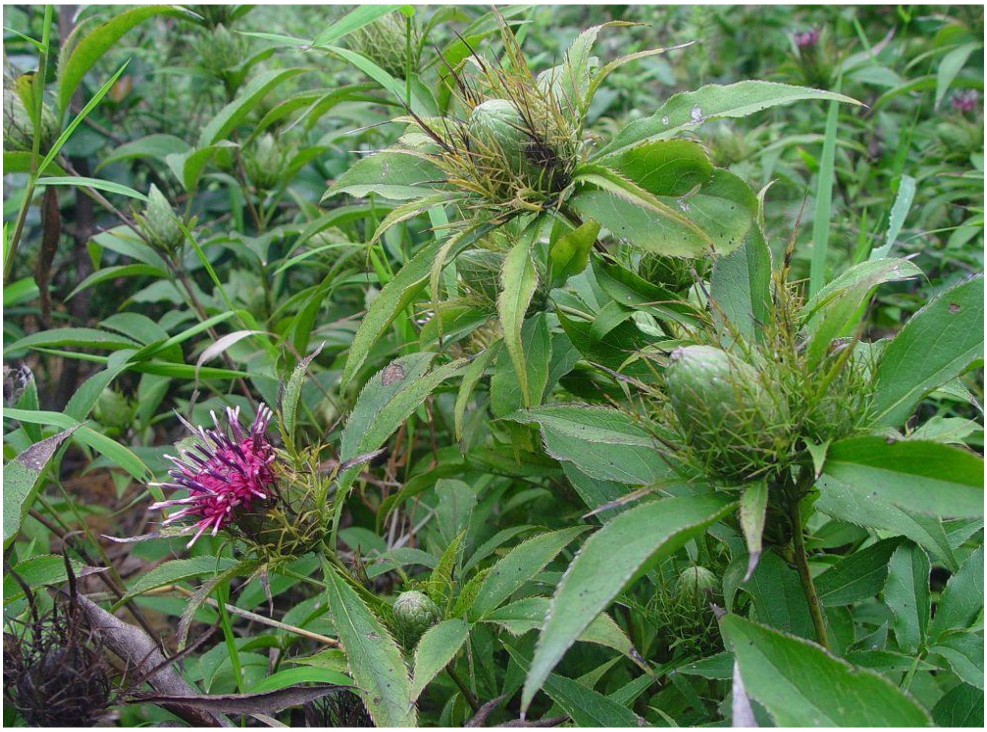

Contributed by Jian Lin, Oct. 11, 2012, Yaowang Mountain, Changzhou University of Chinese medicine, Guangzhou, China, 510006.

Figure S1-2. Dried Rhizoma *Atractylodes macrocephala.*

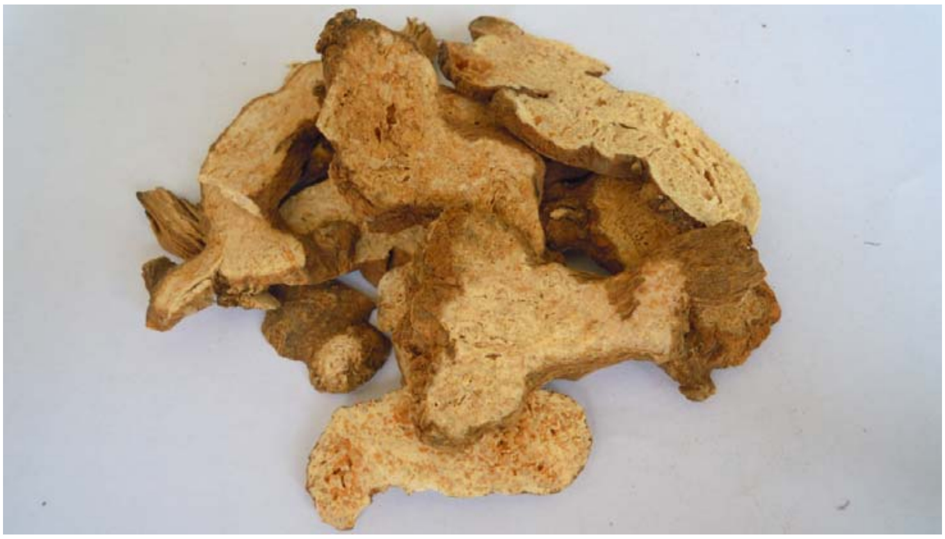

Contributed by Jing Lin, Oct. 11, 2012, Changzhou University of Chinese Medicine, Guangzhou, China, 510006.

Part 2. The calculations of total phenolics, total flavonoids, total sugars and total saponins.

Figure S2-1. The standard curve of pyrogallol ($y = 45.225x + 0.0309$, R = 0.9985).

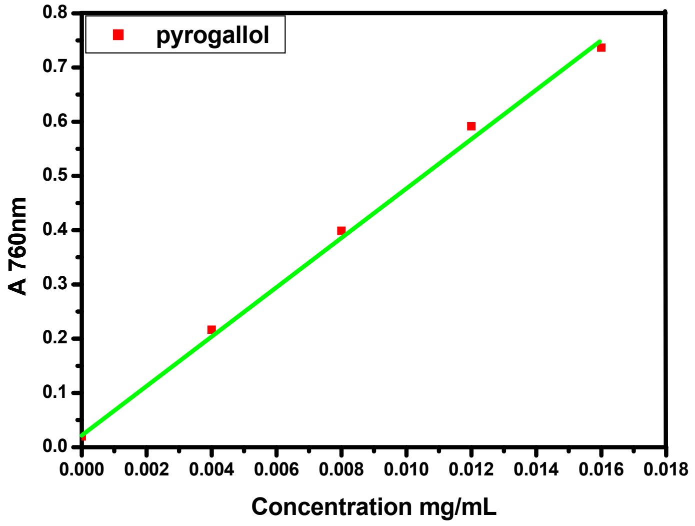

Table S2-1. The total phenolics contents of five AM extracts.

	PEAM	EAAM	AEAM	95EAM	WAM
A_{760nm}	0.338	0.463	0.299	0.221	0.052
	0.338	0.465	0.299	0.221	0.053
	0.338	0.465	0.301	0.220	0.054
Content (mg pyrogallol/g)	15.31695	20.97008	13.64363	10.02563	2.3826
	15.31695	21.06053	13.64363	10.02563	2.427825
	15.31695	21.06053	13.55318	9.9804	2.47305
Mean ± SD	15.32 ± 0.00 [d]	21.03 ± 0.052 [e]	13.61 ± 0.052 [c]	10.01 ± 0.026 [b]	2.43 ± 0.045 [a]

PEAM, petroleum ether extract of Rhizoma *Atractylodes macrocephala*. EAAM, ethyl acetate extract of Rhizoma *Atractylodes macrocephala*. AEAM, absolute ethanol extract of Rhizoma *Atractylodes macrocephala*. 95EAM, 95% ethanol extract of Rhizoma *Atractylodes macrocephala*. WAM, water extract of Rhizoma *Atractylodes macrocephala*.

Figure S2-2. The standard curve of rutin ($y = 11.047x - 0.0105$, R = 0.9988).

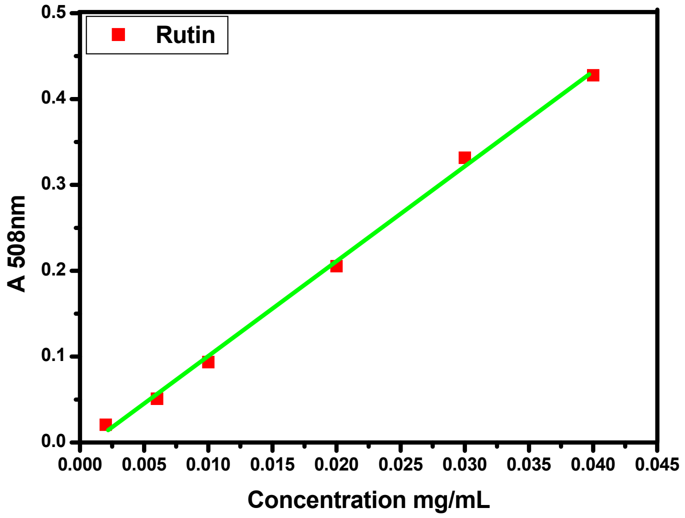

Table S2-2. The total flavonoids contents of five AM extracts.

	PEAM	EAAM	AEAM	95EAM	WAM
A_{760nm}	0.305	0.261	0.046	0.19	0.004
	0.306	0.262	0.046	0.19	0.003
	0.305	0.263	0.046	0.19	0.003
Content (mg rutin/g)	3.358835	2.872767	0.497662	2.08843	0.033688
	3.369882	2.883814	0.497662	2.08843	0.022641
	3.358835	2.894861	0.497662	2.08843	0.022641
Mean ± SD	3.36 ± 0.0064 [d]	2.88 ± 0.011 [c]	0.50 ± 0.00 [b]	2.09 ± 0.00 [c]	0.026 ± 0.064 [a]

PEAM, petroleum ether extract of Rhizoma *Atractylodes macrocephala*. EAAM, ethyl acetate extract of Rhizoma *Atractylodes macrocephala*. AEAM, absolute ethanol extract of Rhizoma *Atractylodes macrocephala*. 95EAM, 95% ethanol extract of Rhizoma *Atractylodes macrocephala*. WAM, water extract of Rhizoma *Atractylodes macrocephala*.

Figure S2-3. The standard curve of glucose ($y = 4.3x - 0.0036$, R = 0.9968).

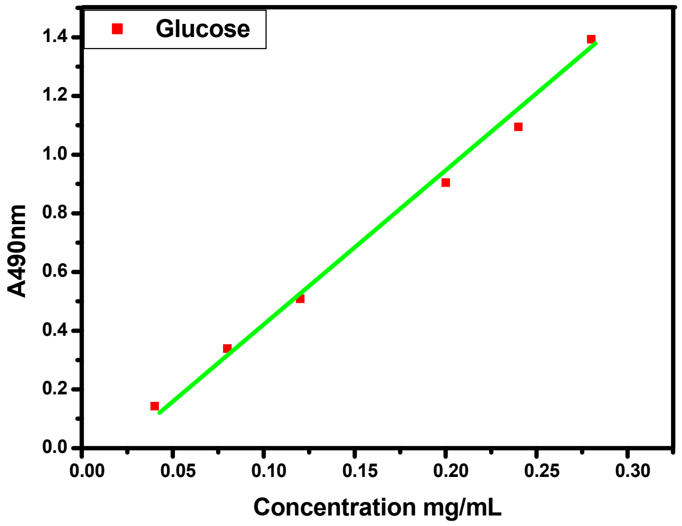

Table S2-3. The total sugars contents of five AM extracts.

	PEAM	EAAM	AEAM	95EAM	WAM
A_{760nm}	0.262	0.357	1.156	1.165	0.828
	0.256	0.361	1.156	1.159	0.830
	0.255	0.360	1.156	1.158	0.827
Content (mg glucose/g)	1.123	1.5315	4.9672	5.0059	3.5568
	1.0972	1.5487	4.9672	4.9801	3.5654
	1.0929	1.5444	4.9672	4.9758	3.5525
Mean ± SD	1.10 ± 0.016 [a]	1.54 ± 0.0090 [a]	4.97 ± 0.00 [c]	4.99 ± 0.016 [c]	3.56 ± 0.0066 [b]

PEAM, petroleum ether extract of Rhizoma *Atractylodes macrocephala*. EAAM, ethyl acetate extract of Rhizoma *Atractylodes macrocephala*. AEAM, absolute ethanol extract of Rhizoma *Atractylodes macrocephala*. 95EAM, 95% ethanol extract of Rhizoma *Atractylodes macrocephala*. WAM, water extract of Rhizoma *Atractylodes macrocephala*.

Figure S2-4. The standard curve of oleanolic acid ($y = 36.894x - 0.0089$, R = 0.9984).

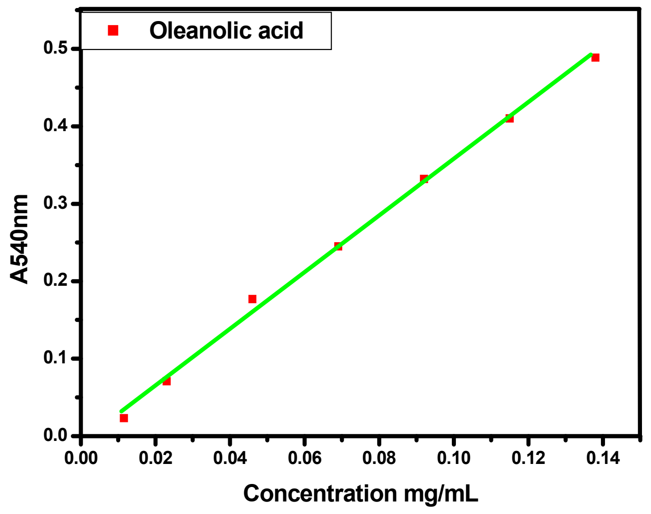

Table S2-4. The total saponins contents of five AM extracts.

	PEAM	EAAM	AEAM	95EAM	WAM
A_{760nm}	0.407	0.165	0.132	0.066	0.066
	0.406	0.162	0.140	0.063	0.075
	0.406	0.168	0.142	0.073	0.071
Content (mg oleanolic acid/g)	15.00696	6.07861	4.861108	2.426104	2.426104
	14.97006	5.967928	5.15626	2.315422	2.75815
	14.97006	6.189292	5.230048	2.684362	2.610574
Mean ± SD	14.98 ± 0.021 [d]	6.08 ± 0.11 [c]	5.08 ± 0.20 [c]	2.48 ± 0.19 [b]	0.026 ± 0.064 [a]

PEAM, petroleum ether extract of Rhizoma *Atractylodes macrocephala*. EAAM, ethyl acetate extract of Rhizoma *Atractylodes macrocephala*. AEAM, absolute ethanol extract of Rhizoma *Atractylodes macrocephala*. 95EAM, 95% ethanol extract of Rhizoma *Atractylodes macrocephala*. WAM, water extract of Rhizoma *Atractylodes macrocephala*.

Part 3. The $1/IC_{50}$ values.

Table S3-1. The $1/IC_{50}$ values of five AM extracts in various antioxidant assays.

	**PEAM**	**EAAM**	**AEAM**	**95EAM**	**WAM**
•OH scavenging	0.00238	0.00712	0.00953	0.00516	0.00280
•O_2^- scavenging	0.00081	0.00143	0.00090	0.00093	0.00046
Fe^{2+} chelating	0.00701	0.00329	0.00184	0.00327	0.00143
Cu^{2+} chelating	0.00741	0.00391	0.00098	0.00197	0.00015
DPPH• scavenging	0.00069	0.00418	0.00288	0.00224	0.00093
$ABTS^+$• scavenging	0.00187	0.01278	0.00714	0.00817	0.00212
Fe^{3+} reducing	0.00226	0.00507	0.00243	0.00238	0.00150
Cu^{2+} reducing	0.00727	0.01201	0.00622	0.00748	0.00360

PEAM, petroleum ether extract of Rhizoma *Atractylodes macrocephala*. EAAM, ethyl acetate extract of Rhizoma *Atractylodes macrocephala*. AEAM, absolute ethanol extract of Rhizoma *Atractylodes macrocephala*. 90EAM, 95% ethanol extract of Rhizoma *Atractylodes macrocephala*. WAM, water extract of Rhizoma *Atractylodes macrocephala*. IC_{50} values are listed in Table 1 in the main text.

Part 4. The HPLC analysis for caffeic acid and ferulic acid.

Figure S4-1. The HPLC profiles of standard caffeic acid and ferulic acid (**A**), PEAM (**B**), EAAM (**C**), AEAM (**D**), 95EAM (**E**), and WAM (**F**).

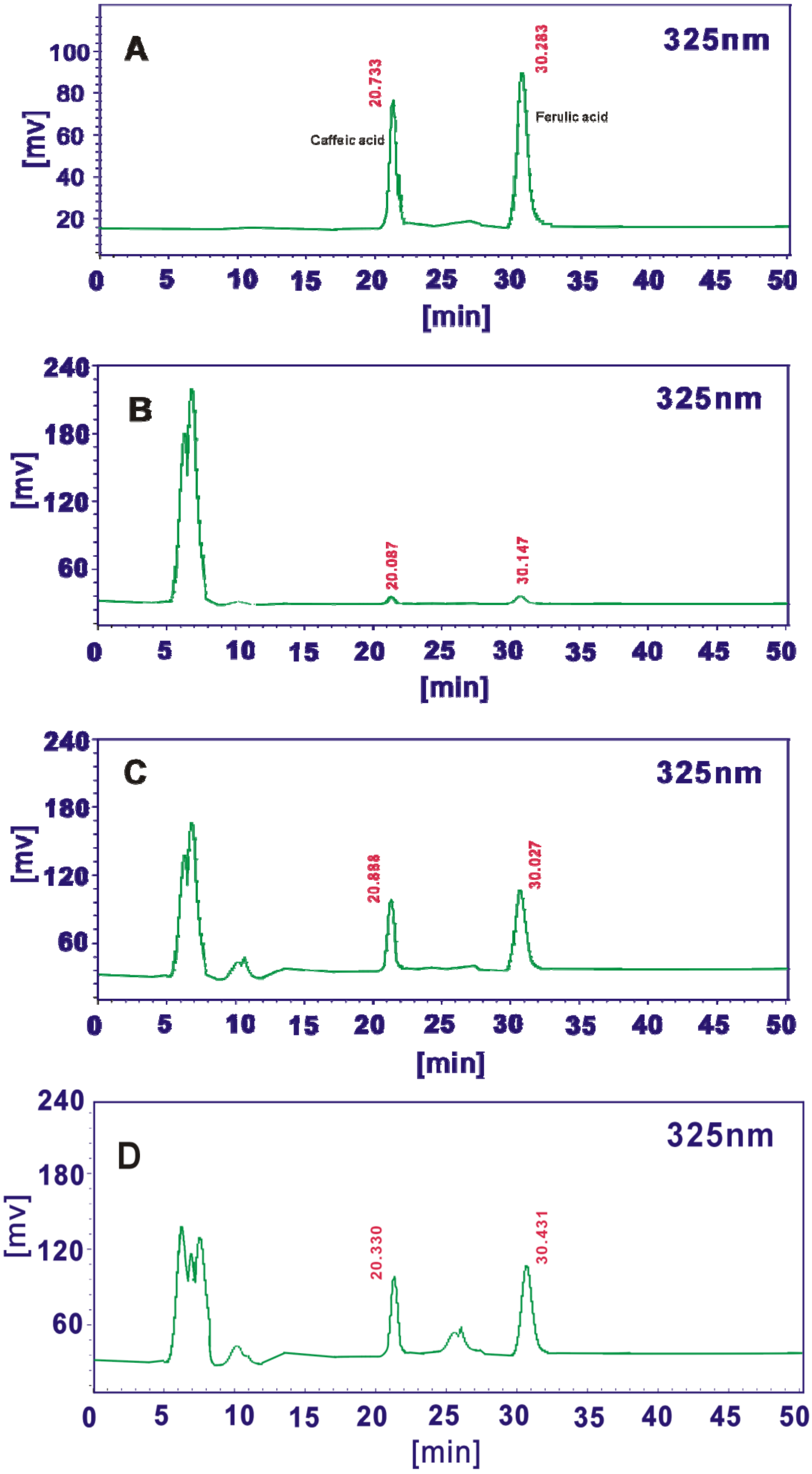

Figure S4-1. *Cont.*

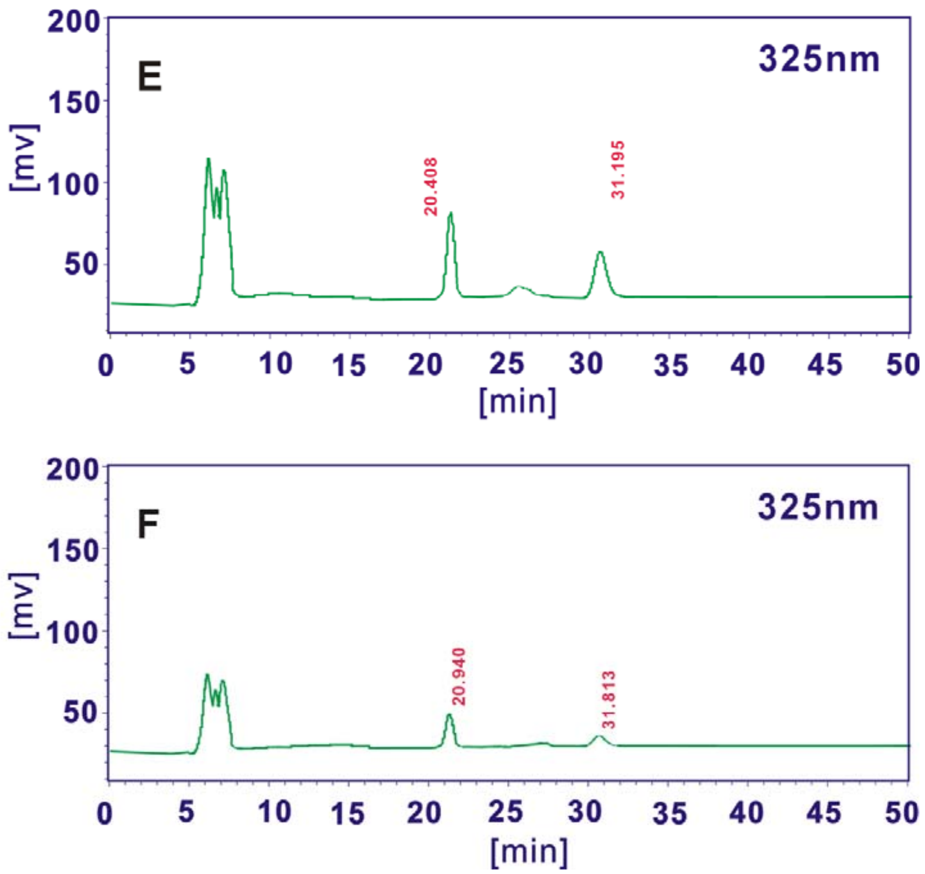

Syltech P510 system (Los Angeles, CA, USA), Dikma Diamonsil C_{18} (250 mm × 4.6 mm, 5 μm) column, methanol:acetonitrile:5% acetate acid (24:6:70), 0.5 mL/min flow rate, 10 μL injection, 325 nm wavelength. PEAM, petroleum ether extract of Rhizoma *Atractylodes macrocephala*. EAAM, ethyl acetate extract of Rhizoma *Atractylodes macrocephala*. AEAM, absolute ethanol extract of Rhizoma *Atractylodes macrocephala*. 95EAM, 95% ethanol extract of Rhizoma *Atractylodes macrocephala*. WAM, water extract of Rhizoma *Atractylodes macrocephala*.

Table S4-1. The retention time, peak area of caffeic acid and ferulic acid in five extracts from Rhizoma *Atractylodes macrocephala*.

	Compound	Retention time/min	Peak area
PEAM	caffeic acid	20.087	5530.33 ± 684.22
	ferulic acid	30.147	7768.53 ± 584.85
EAAM	caffeic acid	20.888	15863.98 ± 1254.02
	ferulic acid	30.027	22358.69 ± 2001.20
AEAM	caffeic acid	20.330	6466.64 ± 655.75
	ferulic acid	30.431	8878.73 ± 784.76
95EAM	caffeic acid	20.408	5990.33 ± 94.99
	ferulic acid	31.195	7968.53 ± 324.90
WAM	caffeic acid	20.940	4333.34 ± 194.72
	ferulic acid	31.813	5013.33 ± 424.87

PEAM, petroleum ether extract of Rhizoma *Atractylodes macrocephala*. EAAM, ethyl acetate extract of Rhizoma *Atractylodes macrocephala*. AEAM, absolute ethanol extract of Rhizoma *Atractylodes macrocephala*. 95EAM, 95% ethanol extract of Rhizoma *Atractylodes macrocephala*. WAM, water extract of Rhizoma *Atractylodes macrocephala*.

Part 5. The HPLC analysis for protocatechuic acid.

Figure S5-1. The HPLC profiles of (**A**) standard protocatechuic acid; and (**B**) petroleum ether extract from Rhizoma *Atractylodes macrocephala* (PEAM); (**C**) ethyl acetate extract from Rhizoma *Atractylodes macrocephala* (EAAM); (**D**) absolute ethanol extract from Rhizoma *Atractylodes macrocephala* (AEAM); (**E**) 95% ethanol extract from Rhizoma *Atractylodes macrocephala* (95EAM); (**F**) water extract from Rhizoma *Atractylodes macrocephala* (WAM).

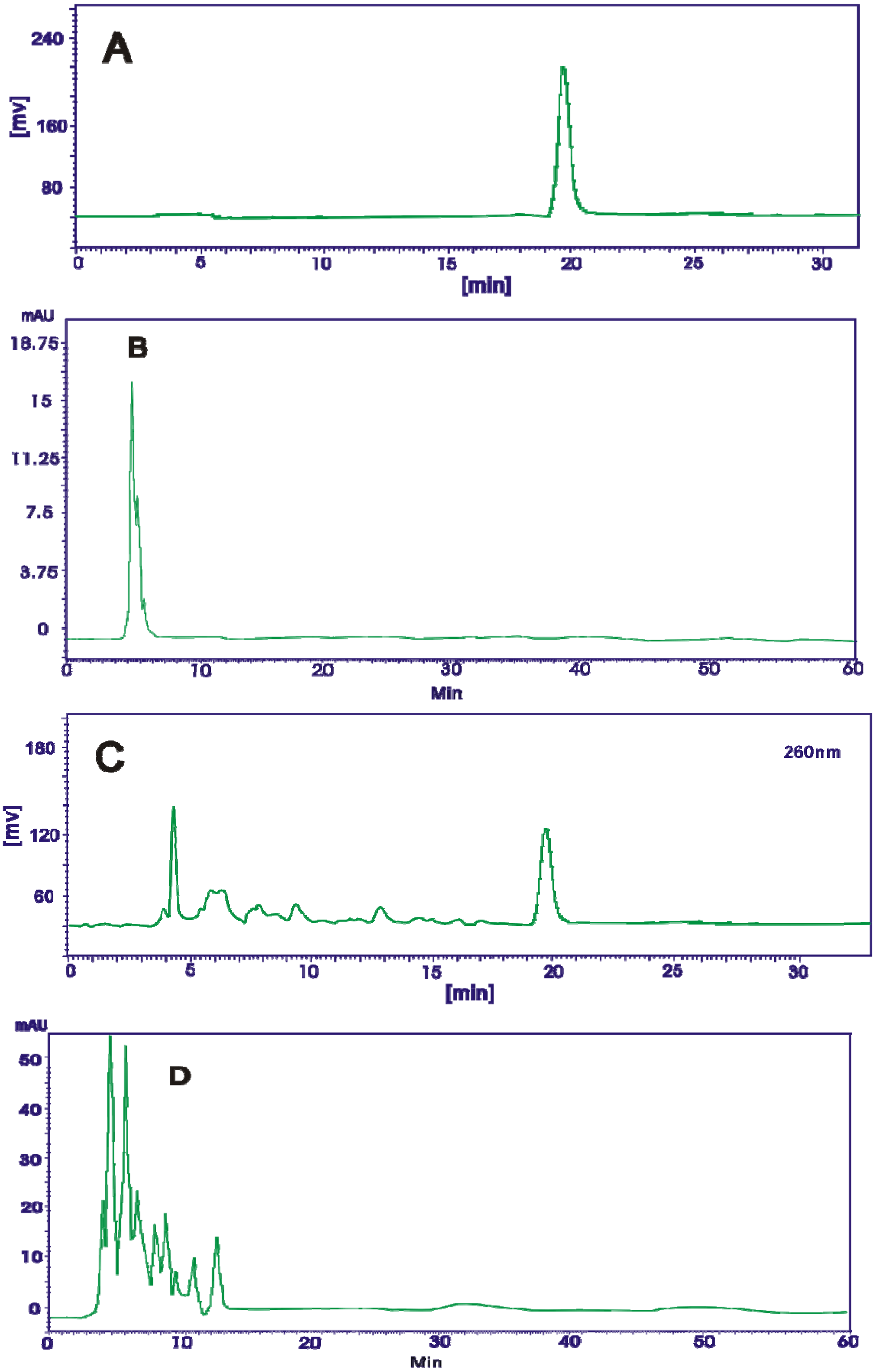

Figure S5-1. *Cont.*

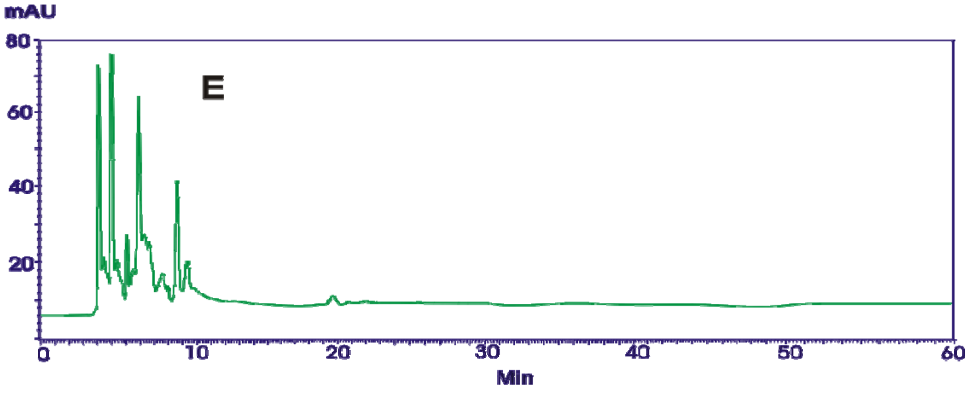

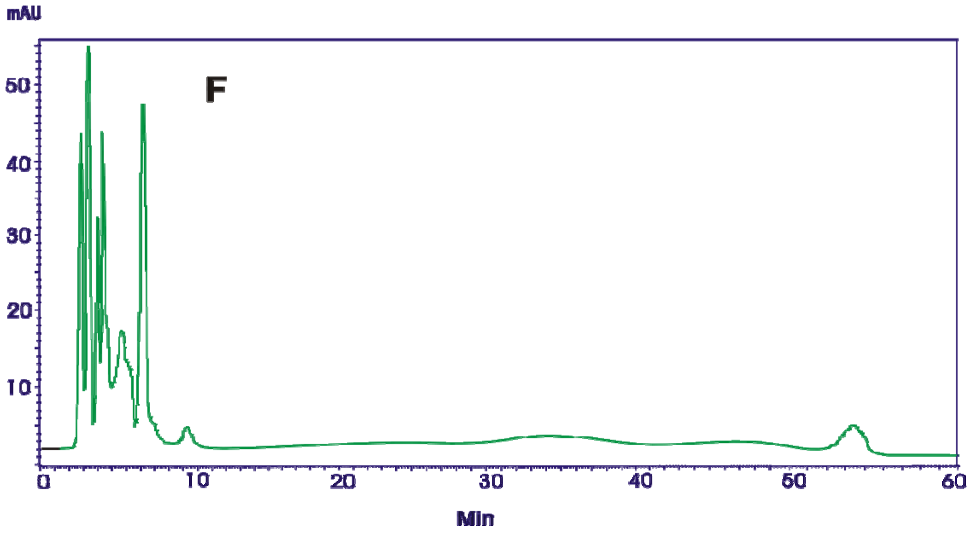

Syltech P510 system (Los Angeles, CA, USA), Dikma Diamonsil C_{18} (250 mm × 4.6 mm, 5 μm) column, 1.0 mL/min flow rate, methanol/acetonitrile/5% acetate acid (12:3:10), 10 μL injection, 60 nm wavelength.

Table S5-1. The retention times and peak areas of protocatechuic acid in five AM extracts.

	**Retention time/min**	**Peak area ***
PEAM	**20.8**	**0**
EAAM	**20.8**	**463523 ± 44277.3**
AEAM	**20.8**	**0**
95EAM	**20.8**	**51570.5 ± 714.89**
WAM	**20.8**	**0**

* The values are expressed as Mean ± SD (*n* = 3). PEAM, petroleum ether extract of Rhizoma *Atractylodes macrocephala*. EAAM, ethyl acetate extract of Rhizoma *Atractylodes macrocephala*. AEAM, absolute ethanol extract of Rhizoma *Atractylodes macrocephala*. 95EAM, 95% ethanol extract of Rhizoma *Atractylodes macrocephala*. WAM, water extract of Rhizoma *Atractylodes macrocephala*.

Part 6. The correlation graphs (56 in total).

Figure S6-1. The correlation graphs between total phenolics and antioxidant levels. (**A**) for •OH assay; (**B**) for $\bullet O_2^-$ assay; (**C**) for Fe^{2+} chelating ability assay; (**D**) for Cu^{2+} chelating ability assay; (**E**) for DPPH assay; (**F**) for ABTS assay; (**G**) for Fe^{3+} reducing power assay; (**H**) for Cu^{2+} reducing power assay.

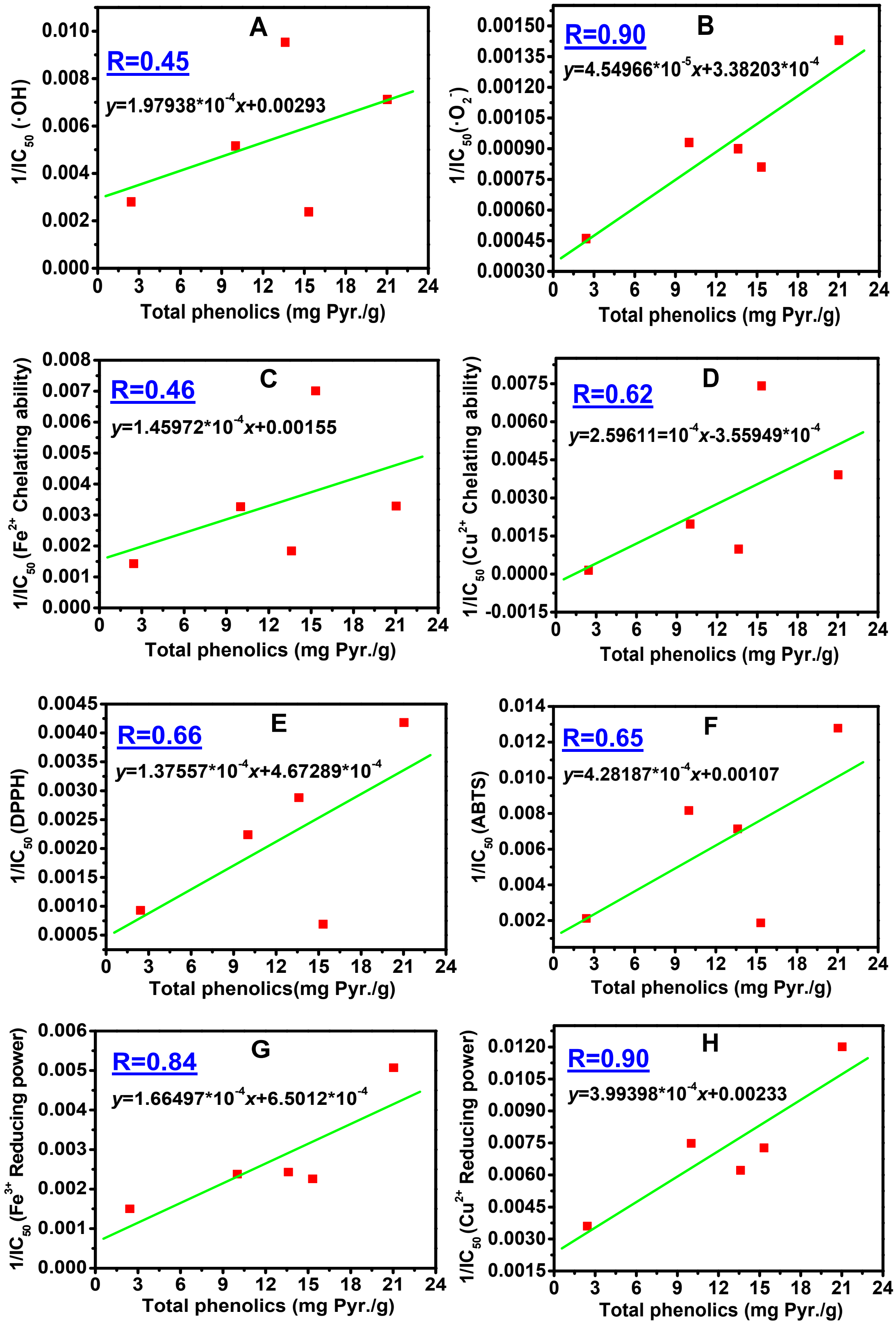

Figure S6-2. The correlation graphs between total flavone and antioxidant levels. (**A**) for •OH assay; (**B**) for $•O_2^-$ assay; (**C**) for Fe^{2+} chelating ability assay; (**D**) for Cu^{2+} chelating ability assay; (**E**) for DPPH assay; (**F**) for ABTS assay; (**G**) for Fe^{3+} reducing power assay; (**H**) for Cu^{2+} reducing power assay.

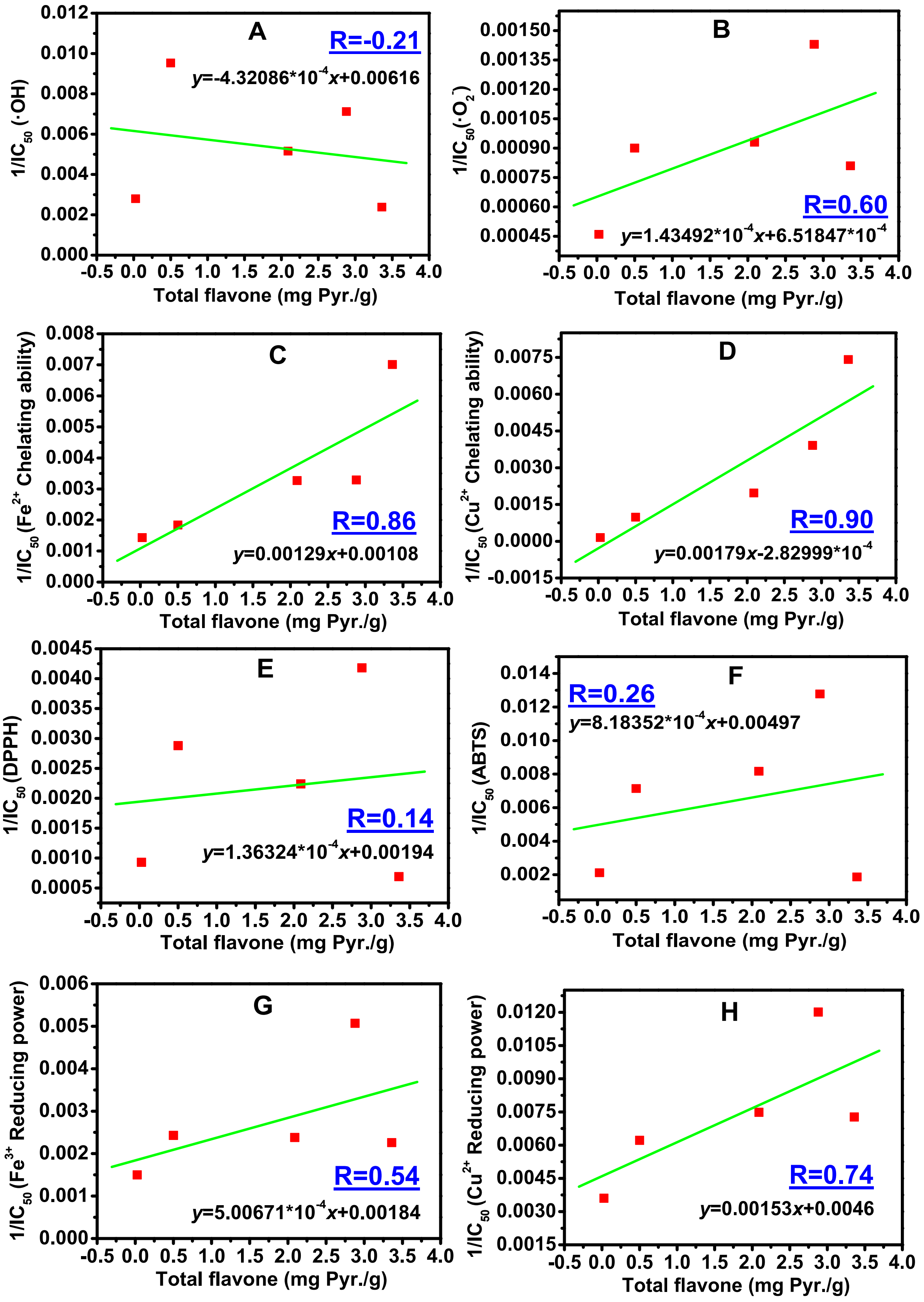

Figure S6-3. The correlation graphs between total sugars and antioxidant levels. (**A**) for •OH assay; (**B**) for $•O_2^-$ assay; (**C**) for Fe^{2+} chelating ability assay; (**D**) for Cu^{2+} chelating ability assay; (**E**) for DPPH assay; (**F**) for ABTS assay; (**G**) for Fe^{3+} reducing power assay; (**H**) for Cu^{2+} reducing power assay.

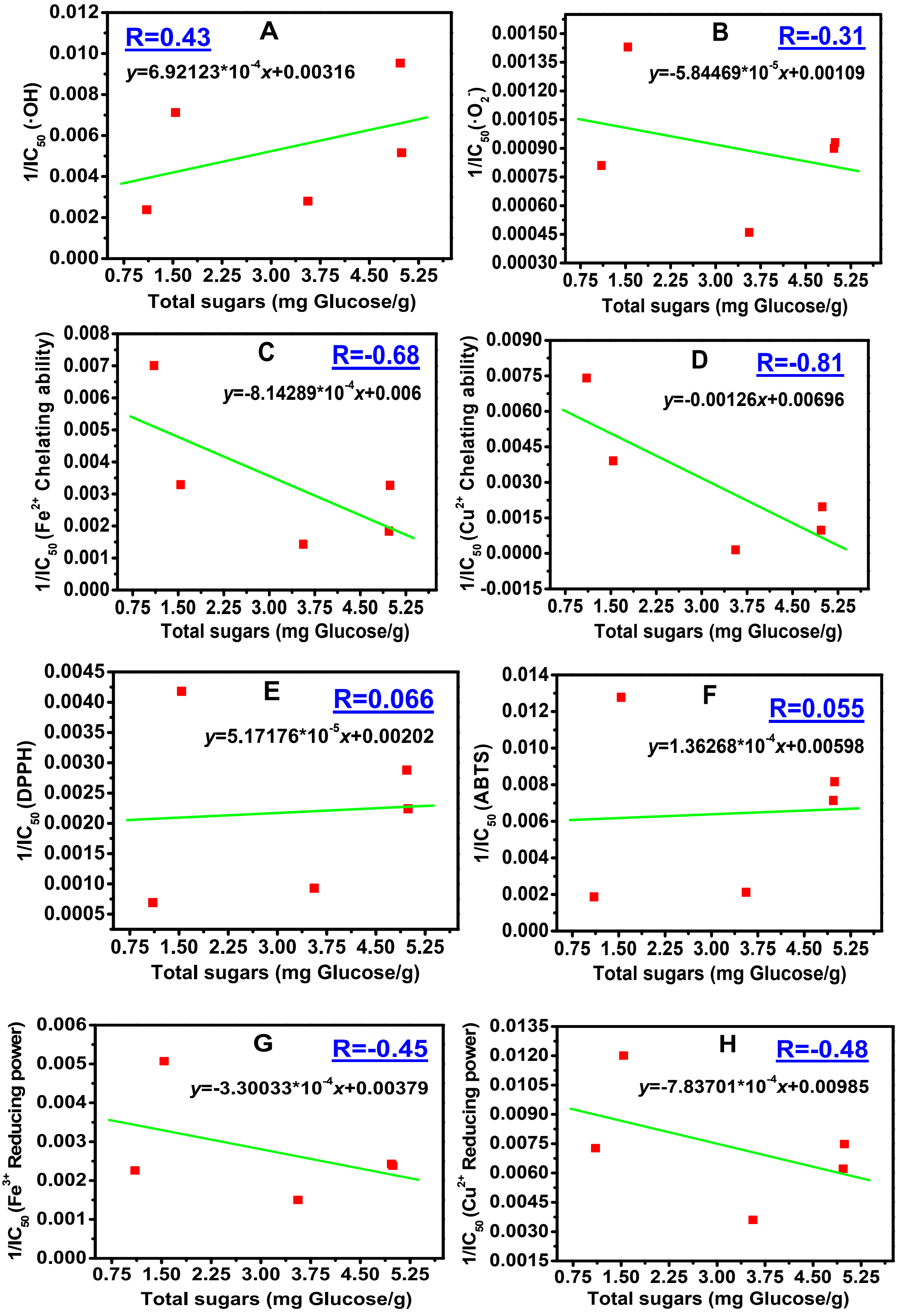

Figure S6-4. The correlation graphs between total saponins and antioxidant levels. (**A**) for •OH assay; (**B**) for $•O_2^-$ assay; (**C**) for Fe^{2+} chelating ability assay; (**D**) for Cu^{2+} chelating ability assay; (**E**) for DPPH assay; (**F**) for ABTS assay; (**G**) for Fe^{3+} reducing power assay; (**H**) for Cu^{2+} reducing power assay.

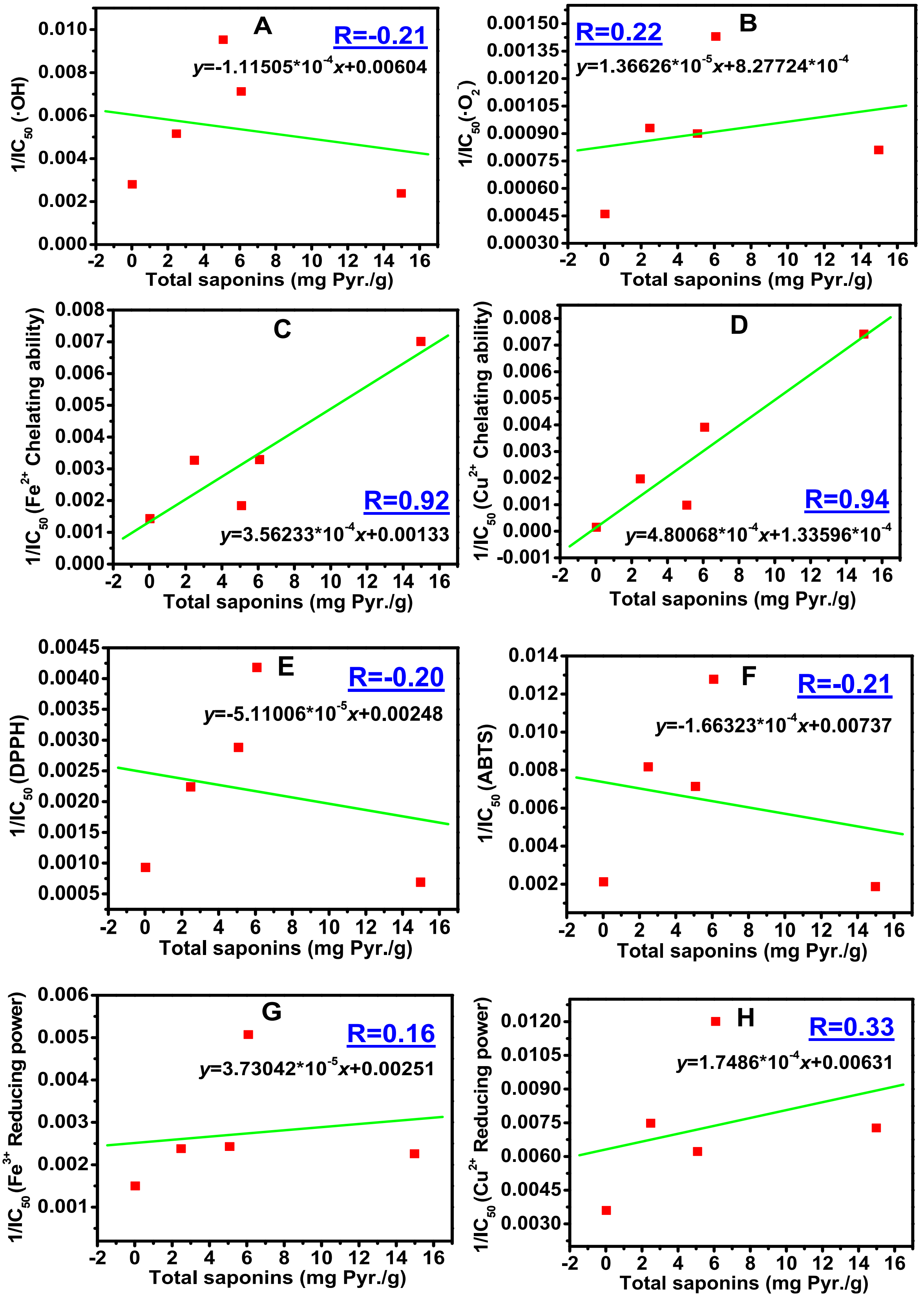

Figure S6-5. The correlation graphs between caffeic acid and antioxidant levels. (**A**) for •OH assay; (**B**) for $•O_2^-$ assay; (**C**) for Fe^{2+} chelating ability assay; (**D**) for Cu^{2+} chelating ability assay; (**E**) for DPPH assay; (**F**) for ABTS assay; (**G**) for Fe^{3+} reducing power assay; (**H**) for Cu^{2+} reducing power assay.

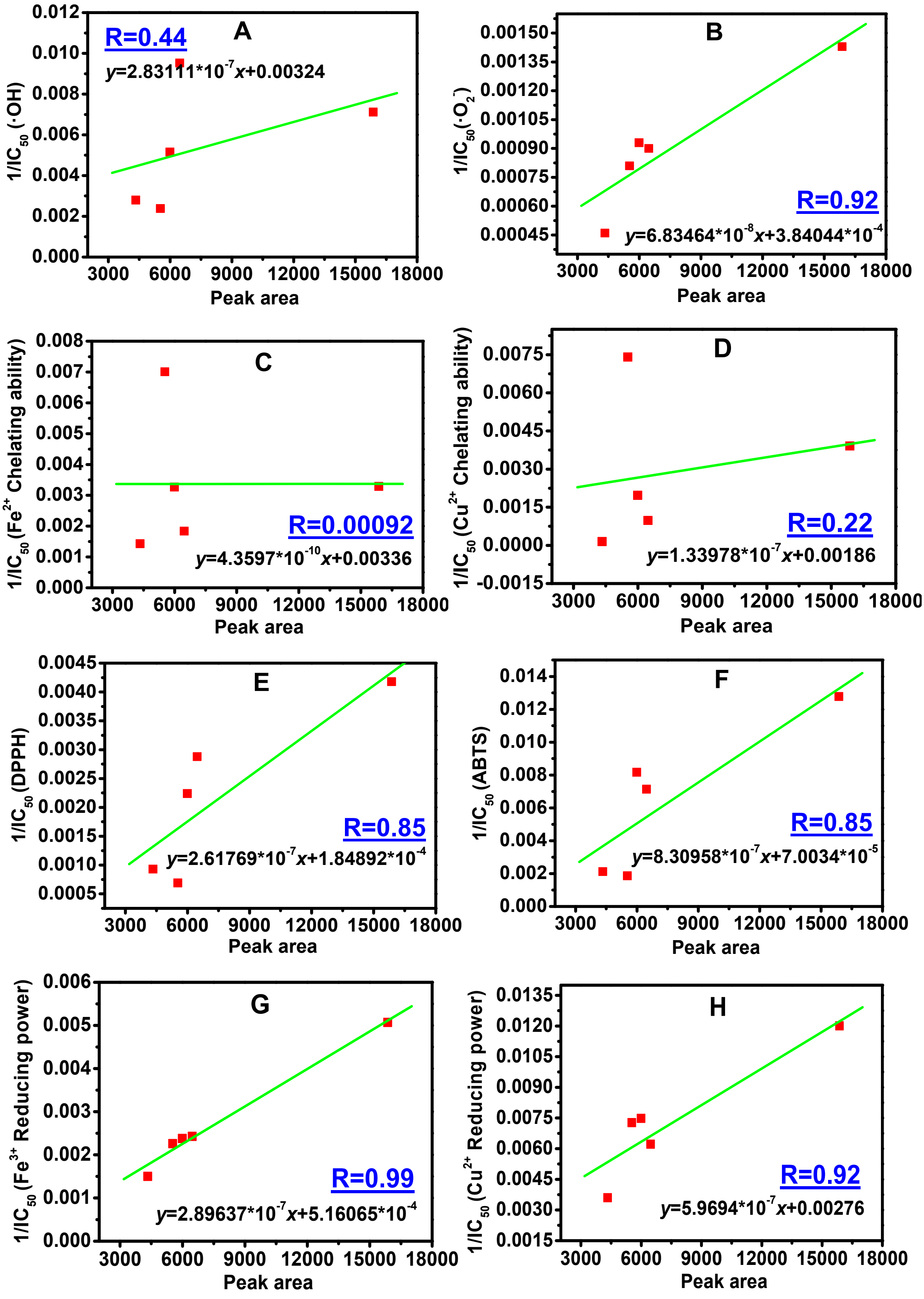

Figure S6-6. The correlation graphs between ferulic acid and antioxidant levels. (**A**) for •OH assay; (**B**) for •O_2^- assay; (**C**) for Fe^{2+} chelating ability assay; (**D**) for Cu^{2+} chelating ability assay; (**E**) for DPPH assay; (**F**) for ABTS assay; (**G**) for Fe^{3+} reducing power assay; (**H**) for Cu^{2+} reducing power assay.

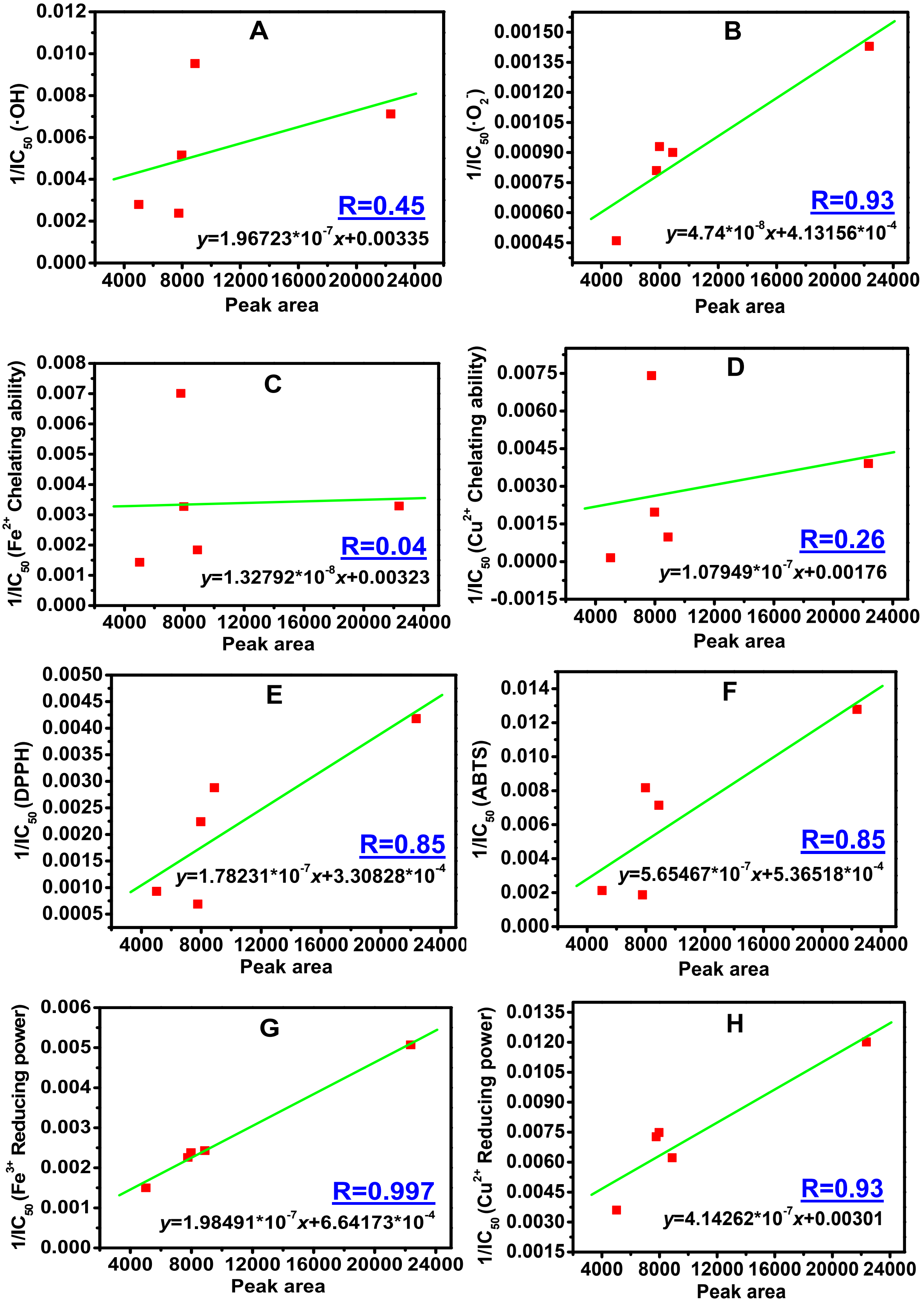

Figure S6-7. The correlation graphs between protocatechuic acid and antioxidant levels. (**A**) for •OH assay; (**B**) for $•O_2^-$ assay; (**C**) for Fe^{2+} chelating ability assay; (**D**) for Cu^{2+} chelating ability assay; (**E**) for DPPH assay; (**F**) for ABTS assay; (**G**) for Fe^{3+} reducing power assay; (H) for Cu^{2+} reducing power assay.

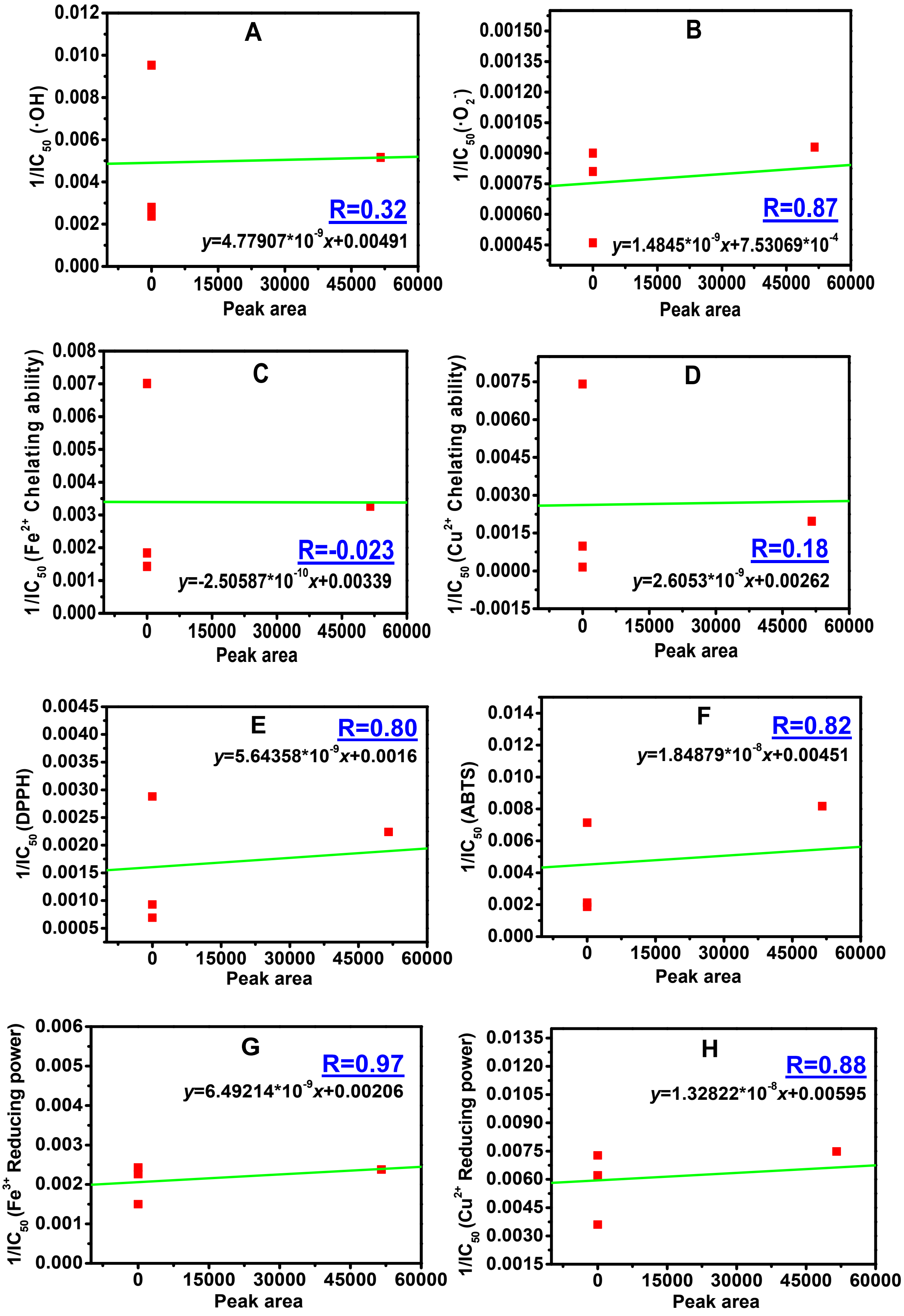

Supplement: Supplementary file 1 [file molecules-17-13457-s001.pdf]
